# Comparison of surgical outcomes between iStent inject W implantation and microhook ab interno trabeculotomy in combination with phacoemulsification in primary open-angle glaucoma patients

**DOI:** 10.3389/fmed.2023.1266532

**Published:** 2023-09-29

**Authors:** Hiromitsu Onoe, Kazuyuki Hirooka, Koji Namiguchi, Shiro Mizoue, Hiroko Hosokawa, Hideki Mochizuki, Naoki Okada, Kana Tokumo, Hideaki Okumichi, Yoshiaki Kiuchi

**Affiliations:** ^1^Department of Ophthalmology and Visual Science, Hiroshima University, Hiroshima, Japan; ^2^Department of Ophthalmology, Ehime University Graduate School of Medicine, Toon, Japan; ^3^Department of Ophthalmology, Minami-Matsuyama Hospital, Matsuyama, Japan; ^4^Kusatsu Eye Clinic, Hiroshima, Japan

**Keywords:** iStent inject W, primary open-angle glaucoma, microhook, phacoemulsification, intraocular pressure

## Abstract

**Purpose:**

To examine primary open-angle glaucoma patients after undergoing combined cataract surgery with microhook ab interno trabeculotomy (μLOT-Phaco) or iStent inject W implantation (iStent-Phaco), and then evaluate the surgical outcomes after a minimum of 6 months of follow-up.

**Methods:**

Between October 2020 and July 2022, 39 μLOT-Phaco eyes and 55 iStent-Phaco eyes that underwent surgery were evaluated in this retrospective, multicenter comparative case series. Data that included preoperative and postoperative intraocular pressure (IOP), number of glaucoma medications, and occurrence of complications were collected from medical records and then examined. Surgical failure was defined as patients exhibiting *a* < 20% reduction in the preoperative IOP or an IOP > 18 mmHg on two consecutive follow-up visits, or when patients were required to undergo reoperation. Success rates were determined based on a Kaplan–Meier survival analysis.

**Results:**

At 3, 6 and 12 months postoperatively, there was a significant postoperative reduction in the IOP (*p* < 0.001) and in the medications scores (*p* < 0.001) for both of the groups. In the μLOT-Phaco and iStent-Phaco groups, the probabilities of success at 6 and 12 months were 55.3 and 45.5%, and 48.4 and 45.5% (*p* = 0.38; log-rank test), respectively. In the iStent-Phaco group, there was a significant decrease in the hyphema.

**Conclusion:**

Comparable surgical outcomes occurred for both the μLOT and iStent inject W procedures.

## Introduction

Glaucoma is a leading cause of irreversible blindness worldwide (1). The use of medications or surgery to lower the intraocular pressure (IOP) are the only ways that have been shown to decrease the speed of the visual field damage progression (2, 3). The first-line therapy normally used includes glaucoma medications and/or selective laser trabeculoplasty. When the IOP cannot be sufficiently lowered or progression prevented after the utilization of the maximum tolerated medical and/or laser treatment, surgery is typically performed.

In mild to moderate glaucoma patients, the interventions available for surgeons have been greatly augmented through the implementation of minimally invasive glaucoma surgery (MIGS) procedure. The iStent trabecular micro-bypass (containing one stent) was the original MIGS device approved by the United Stated Food and Drug Administration (FDA), with the FDA then approving the second-generation iStent inject (containing two stents). As the trabecular meshwork is known to cause resistance to the aqueous humor outflow, in addition to being a major contributor to elevated IOP in glaucoma patients, these stents were both designed to create pathways through this meshwork (4). The iStent inject W (Glaukos Corporation, San Clemente, CA), which is available in Japan, contains a wide flange at its base and is designed to optimize stent visualization and placement. Another trabecular-based device that works by increasing the aqueous humor outflow through Schlemm’s canal is the Tanito microhook (Inami & Co., Ltd., Tokyo, Japan).

As there has been continuous expansion of the field of MIGS along with the development of newer techniques, it is important that measurements of the surgical outcome between different procedures be undertaken. Thus, the postoperative outcomes of combined cataract surgery with either microhook ab interno trabeculotomy (μLOT-Phaco) or iStent inject W implantation (iStent-Phaco) were compared in primary open-angle glaucoma (POAG) patients in our current study.

## Materials and methods

### Patient selection

Eyes undergoing phacoemulsification cataract extraction combined with either μLOT (μLOT-Phaco) or iStent inject W implantation (iStent-Phaco) between October 2020 and July 2022 were evaluated in this retrospective study in the Japanese prefectures of Hiroshima and Ehime at Hiroshima University Hospital, Ehime University Hospital, Minami-Matsuyama Hospital, and Kusatsu Eye Clinic. This study was in accordance with the principles of the Declaration of Helsinki, and was conducted following approval from the Hiroshima university’s ethics committee.

Patients that were ≥ 18 years of age, and previously diagnosed with POAG were included in the study. However, patients were excluded from the study if they had other glaucoma types or if they had a history of ocular surgery. Furthermore, a 6-month postoperative observation period was required in all patients. When bilateral surgery was performed in a patient, the analysis only utilized the data from the first eye. A suboptimal IOP and/or slow progression of visual field damage in spite of maximal tolerable glaucoma therapy were the most common and similar indications for the surgeries. Another relevant surgery group included patients who were on more than one glaucoma medications without known intolerance requiring the number of glaucoma medications reduction that were scheduled for cataract surgery. Surgeons were responsible for deciding whether to use the iStent inject W implantation or microhook ab interno trabeculotomy.

### Surgical technique

Phacoemulsification was performed using the standard technique. After cataract removal was performed through a temporal corneal incision, intraocular lens was implanted in the capsular bag. Sodium hyaluronate was added to the anterior chamber after the cataract surgery to ensure that there was enhanced visuality of Schlemm’s canal. Subsequently, after tilting both the patient’s head and microscope, the gonio lens was placed on the cornea to visualize the nasal angle. In the iStent-Phaco group, following the identification of Schlemm’s canal, the iStent inject W was implanted as two devices, with the two stents located in the nasal quadrant one to two clock hours apart. After inserting the microhook tip into Schlemm’s canal via the main corneal incision in the μLOT-Phaco group, the microhook was circumferentially moved to the 4 clock hour position (nasal quadrant) in order to incise both the trabecular meshwork and the inner wall of the Schlemm’s canal. Antibacterial and anti-inflammatory topical medications were prescribed for each of the patients. All of the glaucoma medications were stopped at the time of the surgery, then added in the postoperative follow-up visits performed at the discretion of the surgeon.

### Outcome measures

Surgical failure was based on IOP criteria, was defined as the primary outcome. The results of the Kaplan–Meier survival analysis with the two criteria were used to determine the success of the procedure. Surgical failure was defined as *a* < 20% reduction in the preoperative IOP or an IOP > 18 mmHg on two consecutive study visits, or when for a patient required a reoperation. However, as it has been reported that there are postoperative IOP fluctuations after trabeculotomy (5), IOPs that met the above criteria for up to 3 months after surgery were not considered to be surgical failures. The number of glaucoma medications used, the presence of any postoperative complications and the mean IOP were all considered to be secondary outcomes. For the mean number of glaucoma medications, 1 point was assigned for each glaucoma eye drop, while 2 points were assigned for the combination eye drops.

### Statistical analysis

Statistical analyses were performed using JMP software (version 16; SAS Inc., Cary, NC). An independent *t*-test and a chi-square test were used to analyze the clinical backgrounds of the subjects. A chi-square test was also used to analyze the differences of the postoperative complications. Kaplan–Meier survival curves were used to analyze the probability of success, with the results compared between the groups using a log-rank test. For continuous variables, the Anderson-Darling test was used to assess the distribution. Based on the results obtained, difference between preoperative and postoperative values were then assessed by either a paired *t*-test or Wilcoxon signed-rank test. All data are expressed as the mean ± standard deviation. *p* values less than 0.05 were defined as being statistically significant.

## Results

In the μLOT-Phaco and iStent-Phaco groups in this study, evaluated 39 and 55 eyes, respectively. Evaluations of the age, gender, preoperative IOP, and number of preoperative glaucoma medications found no significant differences between both groups (Table 1). There were 18 eyes (47%) in the μLOT-Phaco group and 36 eyes (65%) in the iStent-Phaco group (*p* = 0.08) in patients with normal-tension glaucoma.

**Table 1:** Clinical characteristics at baseline.

	μLOT-Phaco	iStent-Phaco	*p* value
No. eyes	39	55	
Age (years)	72.5 ± 8.1	72.2 ± 9.8	0.88
Gender (M/F)	21/18	26/29	0.53
Preoperative IOP (mmHg)	16.2 ± 4.4	15.0 ± 3.8	0.18
No. IOP-lowering medication	2.2 ± 1.4	2.1 ± 1.4	0.68
Prostanoid receptor agonist	32	48	
β-blocker	20	27	
Carbonic anhydrase inhibitor	20	22	
α2 agonist	15	14	
ROCK inhibitor	5	4	
Axial length (mm)	25.1 ± 2.1	25.1 ± 2.6	0.93
Central cornea thickness (μm)	507.7 ± 37.7	510.5 ± 37.1	0.72
Visual field MD (dB)	−9.4 ± 6.7	−9.1 ± 7.0	0.87
Visual field PSD (dB)	8.1 ± 4.2	7.5 ± 4.4	0.39
Whole RNFL thickness (μm)	70.1 ± 12.9	70.4 ± 11.6	0.90
Whole GCC thickness (μm)	74.7 ± 14.7	79.6 ± 26.2	0.35

M, male; F, female; IOP, intraocular pressure; ROCK, rho-kinase; MD, mean deviation; PSD, pattern standard deviation; OCT, optical coherence tomography; RNFL, retinal nerve fiber layer; GCC, ganglion cell complex.

A significant decrease in the IOP was found in both groups when compared to the preoperative levels (Table 2). In the μLOT-Phaco group, the preoperative IOP level was 16.2 ± 4.4 mmHg, while at 3, 6 and 12 months postoperatively, the levels were 12.7 ± 2.9 mmHg, 12.5 ± 2.6 mmHg, and 13.2 ± 3.2 mmHg, respectively. In the iStent-Phaco group, the preoperative IOP level was 15.0 ± 3.8 mmHg, while at 3, 6 and 12 months postoperatively, the levels were 12.4 ± 2.6 mmHg, 12.5 ± 2.3 mmHg, and 13.2 ± 2.4 mmHg, respectively. Significant differences were observed in both groups between the preoperative and postoperative IOP levels at all patient visits. There were no significant differences observed at each visit with regard to the postoperative IOP reduction in these two groups (*p* = 0.34, 0.10, 0.23).

**Table 2:** Differences in postoperative IOP.

		μLOT-Phaco			iStent-Phaco		
	IOP (mmHg)	Change from baseline (%)	*P* value*	IOP (mmHg)	Change from baseline (%)	*P* value*	*P* value** (95% CI)
Baseline	16.2 ± 4.4			15.0 ± 3.8			
Month 3	12.7 ± 2.9	18.4 ± 21.0	<0.001	12.4 ± 2.6	14.4 ± 18.9	<0.001	0.34 (−4.1 ~ 12.6)
Month 6	12.5 ± 2.6	20.4 ± 18.7	<0.001	12.5 ± 2.3	13.8 ± 18.8	<0.001	0.10 (−1.3 ~ 14.5)
Month 12	13.2 ± 3.2	16.2 ± 18.3	<0.001	13.2 ± 2.4	10.0 ± 18.9	<0.001	0.23 (−3.9 ~ 16.3)

IOP, intraocular pressure; CI; confidence interval.

*Calculated using paired *t*-test for IOP between preoperative and postoperative values.

**Calculated using Student’s *t*-test for the % changes from baseline at each visit time between the groups.

In both groups, there was a significant decrease postoperatively in the number of glaucoma medications (Table 3). In the μLOT-Phaco group, the number of preoperative medications was 2.2 ± 1.4, while at 3, 6 and 12 months postoperatively, it was 0.4 ± 1.0, 0.7 ± 1.1, and 0.6 ± 1.0, respectively. In the iStent-Phaco group, the number of preoperative medications was 2.1 ± 1.4, while at 3, 6, and 12 months postoperatively, it was 0.4 ± 0.8, 0.5 ± 0.8, and 0.5 ± 0.8, respectively. In the μLOT-Phaco group, the medication-free rate was 66.7% (26 eyes) and 68.0% (17 eyes) at 6 and 12 months postoperatively, respectively. In the iStent-Phaco group, the medication-free rate was 69.1% (38 eyes) and 69.4% (25 eyes) at 6 and 12 months postoperatively, respectively. The medication-free rate was similar in both groups (*p* = 0.78: 6 months; *p* = 0.90: 12 months). Table 4 shows the category for the postoperative IOP-lowering medications.

**Table 3:** Differences postoperative number of glaucoma medication.

	μLOT-Phaco		iStent-Phaco		
	Number of medications	*P* value*	Number of medications	*P* value*	*P* value**(95% CI)
Baseline	2.2 ± 1.4		2.1 ± 1.4		0.68 (−0.46 ~ 0.71)
Month 3	0.4 ± 1.0	<0.001	0.4 ± 0.8	<0.001	0.98 (−0.35 ~ 0.36)
Month 6	0.7 ± 1.1	<0.001	0.5 ± 0.8	<0.001	0.22 (−0.30 ~ 0.77)
Month 12	0.6 ± 1.0	<0.001	0.5 ± 0.8	<0.001	0.51 (−0.31 ~ 0.62)

IOP, intraocular pressure; CI, confidence interval.

*Calculated using Wilcoxon signed-ranks test for number of medications between preoperative and postoperative values.

**Calculated using Student’s *t*-test for the % changes from baseline at each visit time between the groups.

**Table 4:** Postoperative IOP-lowering medication.

	μLOT-Phaco	iStent-Phaco
Prostanoid receptor agonist	10	15
β-blocker	7	5
Carbonic anhydrase inhibitor	4	5
α2 agonist	4	2
ROCK inhibitor	1	0

IOP, intraocular pressure; ROCK, rho-kinase.

**Figure 1:**
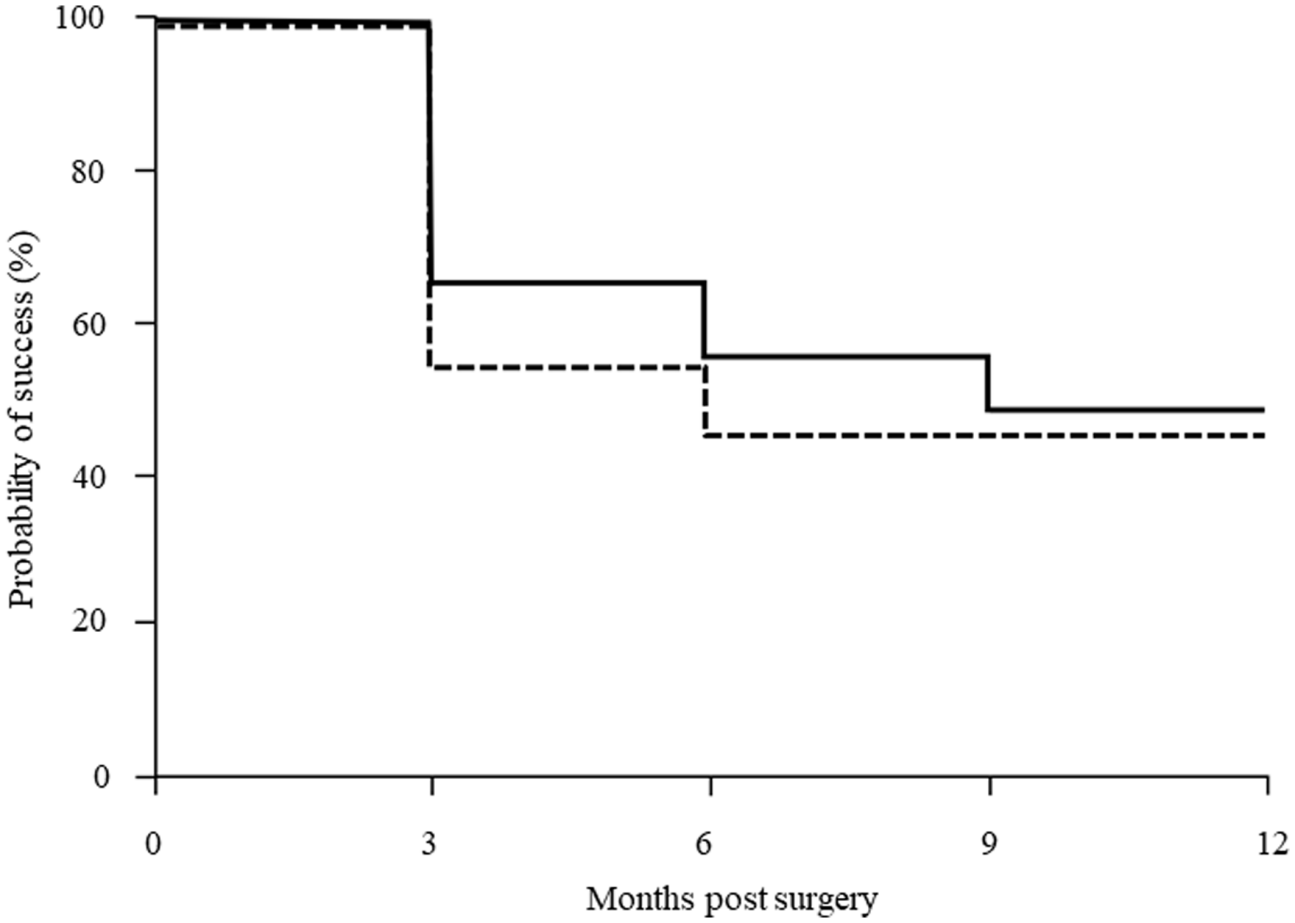
Kaplan–Meier survival analysis success rate of IOP control after μLOT and iStent in combination with phacoemulsification. There were no significant differences observed between two groups (*p* = 0.38, log-rank test). Solid line: μLOT-Phaco group, dashed line: iStent-Phaco group.

The Kaplan–Meier survival curves are seen in Figure 1 for the μLOT-Phaco and iStent-Phaco groups. At 6 and 12 months postoperatively, the survival in the μLOT-Phaco group was 55.3 and 48.4%, while in the iStent-Phaco group it was 45.5 and 45.5%, respectively. Between these two groups, there were no significant differences observed (*p* = 0.38).

Postoperative complications are shown in Table 5. Transient IOP elevation was defined as an elevation of the IOP to ≥30 mmHg within 2 months postoperatively. Hyphema was defined as the presence of the niveau formation. Hyphema was observed in 5 eyes (12.8%) in the μLOT-Phaco group, while in the iStent-Phaco group, 1 eye (1.8%) was found to have hyphema. Analysis indicated that significant differences were noted between the two groups (*p* = 0.03). Transient IOP elevation was observed in 2 eyes (5.1%) of the μLOT-Phaco group. In the μLOT-Phaco group and the iStent-Phaco group, transient IOP elevation occurred in 2 eyes (5.1%) and 2 eyes (3.6%), respectively (*p* = 0.72).

**Table 5:** Postoperative complications.

	μLOT-Phaco	iStent-Phaco	*P* value
Hyphema with niveau	5 (12.8%)	1 (1.8%)	0.03
Transient IOP elevation ≥30 mmHg	2 (5.1%)	2 (3.6%)	0.72

IOP, intraocular pressure.

## Discussion

This study examined μLOT-Phaco and iStent-Phaco, which are two MIGS technologies, in POAG patients by directly comparing relevant clinical data. Comparable surgical outcomes for both μLOT-Phaco and iStent-Phaco were observed at 12 months. During the 12-month follow-up period, there were significant reductions in both the IOP values and glaucoma medications in the μLOT-Phaco and iStent-Phaco groups.

Surgical outcomes have been evaluated between μLOT and iStent combined with cataract surgery (6, 7) and between goniotomy with Kahook Dual Blade (KDB) or iStent combined with cataract surgery (8) in two previous retrospective studies. Equal or less effectiveness was found for iStent procedures with cataract surgery as compared with μLOT or KDB procedures with cataract surgery. However, the IOP-lowering effects and complications of iStent inject W complications as compared to other MIGS procedures have yet to be definitively evaluated. Thus, this current examination of the efficacy of combined phacoemulsification with μLOT and iStent inject W appears to be the first study to compare these procedures. We recently reported that comparable surgical outcomes were found for the use of μLOT and KDB in the combination with cataract surgery (9). Therefore, we assume that comparable surgical outcomes may be obtained for the use of these three different instruments.

When the higher preoperative IOP groups were compared to the lower preoperative IOP groups, a large %IOP reduction was achieved when using the μLOT procedure (10). As our current study found the preoperative IOPs in the μLOT-Phaco and iStent-Phaco groups to be 16.2 mmHg and 15.0 mmHg, respectively, it appears that it would be better to evaluate our current results in line with the similar preoperative IOPs reported in previous papers. In previous studies on open-angle glaucoma patients who underwent the iStent-Phaco procedure, patients who had baseline IOPs of 17.3 mmHg or 17.0 mmHg showed reductions of the IOP of 26.6% or 17.8%, respectively (11, 12). Moreover, these studies reported values of 12.7 mmHg or 14.0 mmHg for the final IOPs. In contrast, procedures in open-angle glaucoma patients who underwent the μLOT-Phaco procedure were found to have a baseline IOP of 16.7 mmHg followed by a 17.8% reduction in the IOP reduction and with a final IOP of 13.6 mmHg (7). The IOP reduction rate and final IOP in the iStent-Phaco and μLOT-Phaco groups in our study were 10.0% and 13.2 mmHg, and 16.2% and 13.2 mmHg, respectively. It should be noted that as compared to the previous study, since we found a lower baseline IOP, this resulted in a tendency for a lower IOP reduction rate in our current study. Even so, as compared to the other previous studies, we found a similar final IOP.

In the μLOT-Phaco group, we found that the hyphema was significantly higher than that observed in the iStent-Phaco group in our current study. Moreover, on postoperative days 1 and 2, the μLOT-Phaco group exhibited a higher postoperative hyphema score as compared to the iStent-Phaco group (13). It has also been reported that there was a significantly higher frequency of layered hyphema in the μLOT group as compared to the iStent group (6). It should be noted, however, that in the current study, the incision of the inner wall of Schlemm’s canal produced by μLOT was approximately one third of the circumference. In contrast, this was a much wider range of incision as compared to that observed for the iStent inject W implantation.

In our current study there were several limitations including the fact that this was a retrospective study. As a result, the assignments of subjects to the treatment groups were not random. Furthermore, we utilized our routine clinical practice to collect the outcome data. It should also be noted that it was up to the surgeon’s discretion to prescribe the IOP-lowering medications at the time of the postoperative follow-up visits. Therefore, to ensure that there is a better collection of data and rigorous comparative evidence, it will be necessary to perform a further randomized and prospective study. Another limitation that needs to be taken into consideration is that there was a relatively low preoperative IOP. Many of the patients that were included in the study had a relatively medically controlled IOP due to being scheduled for the μLOT-Phaco or iStent-Phaco procedures. As a result, there were larger changes in the %IOP reduction in higher preoperative IOP groups when compared to the lower preoperative IOP groups (10). Although it has been recommended that the efficacy of glaucoma surgery be defined based on the combined use of the absolute IOP levels and %IOP reduction (14), the merit of MIGS might be underestimated based on these definitions, especially in eyes having a low preoperative IOP that then undergo surgery.

In conclusion, utilization of the μLOT and iStent inject W procedures resulted in comparable surgical outcomes. Moreover, significant reductions in the IOP were observed when these were combined with cataract surgery, with these POAG patients also showing a reduction in the number of glaucoma medications.

## Data availability statement

The raw data supporting the conclusions of this article will be made available by the authors, without undue reservation.

## Ethics statement

The studies involving humans were approved by the Institutional Review Board of the Hiroshima University. The studies were conducted in accordance with the local legislation and institutional requirements. The participants provided their written informed consent to participate in this study.

## Author contributions

HOn: Writing – original draft, Data curation, Formal analysis, Investigation. KH: Conceptualization, Methodology, Project administration, Writing – original draft, Writing – review & editing. KN: Data curation, Writing – original draft. SM: Data curation, Writing – original draft. HH: Data curation, Writing – original draft. HM: Data curation, Writing – original draft. NO: Project administration, Writing – original draft. KT: Data curation, Writing – original draft. HOk: Data curation, Writing – original draft. YK: Writing – review & editing.
